# Relationship Between Degeneration or Sagittal Balance With Modic Changes in the Cervical Spine

**DOI:** 10.7759/cureus.12949

**Published:** 2021-01-27

**Authors:** Serkan Kitiş, Serdar Çevik, Atilla Kaplan, Hakan Yılmaz, Salim Katar, Serhat Cömert, Ülkün Ünsal

**Affiliations:** 1 Neurosurgery, Bezmialem University, Istanbul, TUR; 2 Neurosurgery, Memorial Şişli Hospital, Istanbul, TUR; 3 School of Health Sciences, Gelişim University, Istanbul, TUR; 4 Department of Radiology, Yalova State Hospital, Yalova, TUR; 5 Radiology, Uşak University, Uşak, TUR; 6 Department of Neurosurgery, Balıkesir Üniversitesi, Balıkesir, TUR; 7 Department of Neurosurgery, Yenimahalle Training and Research Hospital, Ankara, TUR; 8 Department of Neurosurgery, Manisa Şehir Hospital, Manisa, TUR

**Keywords:** cervical spine, modic changes, facet degeneration, disc degeneration, t1 slope, cobb angle, magnetic resonance imaging

## Abstract

Objective: This study evaluates the relationship between degenerative and Modic changes (MCs) in the cervical spine and compares the results with the cervical sagittal balance parameters.

Methods: We retrospectively reviewed 275 patients with neck pain who applied to our outpatient clinic and underwent cervical magnetic resonance imaging (MRI) and cervical anteroposterior (AP)/lateral (Lat) X-ray radiography between January 2016 and January 2018. The clinics, demographic information, and radiological findings of the patients were examined. Modic changes, disc degeneration, and facet degeneration (FD) were examined by cervical MRI, and T1 slope and Cobb angle were measured by cervical AP/Lat X-ray radiography. These results were compared to evaluate their relations with each other.

Results: No relationship between the presence or absence of degenerative changes (Modic changes, facet degeneration, and disc degeneration) and sagittal balance parameters (T1 slope and Cobb angle) was found. However, when each cervical segment was examined separately, facet degeneration at the C4-C5 level and Modic changes at the C3-C4, C4-C5, and C6-C7 levels were statistically significant with the Cobb angles, and the Modic changes at the C3-C4 level and disc degeneration at the C2-C3 level were found to be significant with T1 slope values.

Conclusions: Our findings indicate that MCs increased with decreased cervical curvature, increasing disc and facet degeneration, although the causal mechanisms are not clear.

## Introduction

Neck pain is an important factor affecting daily activities. Although the leading causes of neck pain include disc herniation, disc degeneration (DD), spinal stenosis, and muscle spasm, it is often assumed that neck pain is multifactorial. In addition, most patients with neck pain have radiological findings similar to those in a healthy population [1].

Studies have reported on the relationship between degenerative spine changes such as DD, disc displacement, Schmorl nodes, and facet degeneration (FD) with Modic changes (MCs) in the cervical spine and pain [2-5]. MCs, which have been emphasized in studies on low back pain, have been increasingly associated with neck pain [6-8].

MC is defined as signal changes in the subchondral bone marrow on magnetic resonance images (MRI) in patients with degenerative spine. They are classified into three types according to the signal changes seen in T1- and T2-weighted images in MRI. Type 1 is hypointense on T1-weighted MRI and hyperintense on T2-weighted MRI and is detected in inflamed areas. Type 2 is hyperintense on T1- and T2-weighted MR images and is detected in areas with fatty degeneration. Type 3 is detected in areas with hypointense and sclerosis on T1- and T2-weighted MR images [9].

Cervical segmental instability frequently occurs simultaneously with MC. It is thought that cervical instability may accelerate the degeneration of the normal cervical spine structure. However, it is unknown whether the two diseases are related [10]. Likewise, it was thought that the sagittal profile and individual segmental features of the cervical spine may be responsible for the clinical picture associated with neck pain [1].

In this study, we evaluated the relationship between degenerative changes in the cervical spine (FD and DD) and MCs and compared the results with cervical sagittal balance parameters.

## Materials and methods

Patient data, study design, and study criteria

The data analyzed in this study were obtained from a university hospital over 24-month period (Jan 2016 - Jan 2018) and were reviewed to identify all patients who had neck pain.

All patients' radiological imaging (cervical MRI and cervical anteroposterior (AP)/lateral (Lat) X-ray radiography) and clinical data were obtained using the hospital data system. The MRI scans of these patients were then assessed to evaluate disc degeneration, facet degeneration, and the presence and type of Modic changes in the cervical spine. Sagittal spine curvature was also measured with cervical AP/Lat X-ray radiography. The study was approved by the local ethics committee (20/382).

Inclusion criteria: patients between the ages of 20-75, who had neck pain and had cervical MRI and cervical AP/Lat X-ray radiography due to neck pain were included in our study.

Exclusion criteria: patients with any pathological lesions, such as recent spinal surgery, spinal infections or tumors, inflammatory spondyloarthropathy, congenital or acquired block vertebrae, metabolic diseases, or chronic inflammatory conditions were excluded in our study.

Radiological evaluation

Standard sagittal T1- and T2-weighted axial images were obtained using 5-mm contiguous slices in all directions and an in-plane resolution of 1 mm^2^ or less. All MR images were analyzed by a radiologist who was blinded to the clinical features of the patients. T1- and T2-weighted sagittal images were evaluated, and the MCs were reported as previously described in the literature (MC type I, MC type II, MC type III, MC type I/II, MC type II/III, and MC type I/II/III). DD was determined using sagittal MR images from the C2 to the C7 using Miyazaki’s grading system (Table 1) [11]. Furthermore, cervical FD was determined bilaterally from the C2 to the C7 using axial, sagittal, or coronal MR images, and the patients were divided into two groups: FD (+) and FD (-).

**Table 1:** Miyazaki’s grading system [11]

Grade	Nucleus signal intensity	Nucleus structure	Distinction of nucleus and annulus	Disc height
1	Hyperintense	Homogeneous, white	Clear	Normal
2	Hyperintense	Inhomogeneous with horizontal band, white	Clear	Normal
3	Intermediate	Inhomogeneous, gray to black	Unclear	Normal to decreased
4	Hypointense	Inhomogeneous, gray to black	Lost	Normal to decreased
5	Hypointense	Inhomogeneous, gray to black	Lost	Collapsed

The cervical curvature was assessed using the Cobb method between the C2 and C7 based on the angle, which was measured by cervical AP/Lat X-ray radiography two lines perpendicular to the inferior endplate of the C2 and the superior endplate of the C7, respectively. T1 slope was also measured by cervical AP/Lat X-ray radiography as the angle between a horizontal line and the upper endplate of T1.

Statistical analysis

All analyses were performed using Statistical Package for Social Sciences (SPSS) version 20 (IBM Corp., Armonk, NY, USA). To evaluate the data, frequency, percentage, mean, standard deviation, and crosstab analysis, in addition to descriptive statistical methods, were used to determine the demographic characteristics of the patients. Normally distributed data were evaluated using an independent sample t-test. Pearson’s correlation test was used for correlation analysis. P values less than 0.05 were considered statistically significant.

## Results

**Figure 1:**
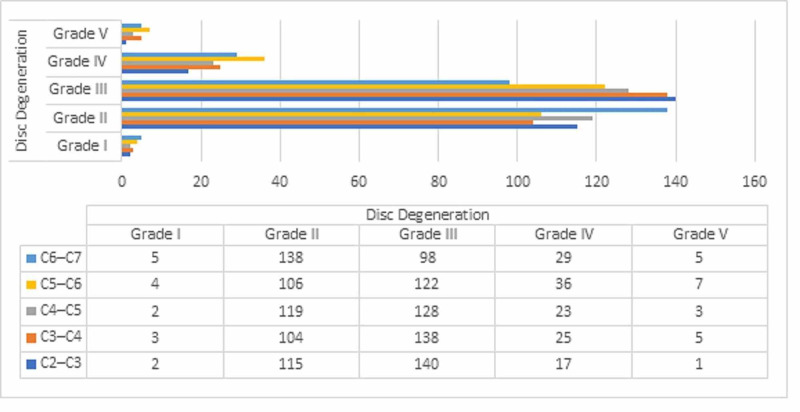
Prevalence of Disc Degeneration at each vertebral level.

Of the 275 patients, 166 were women and 109 were men, and their mean age was 41.1 ± 12.18 years (range, 20-75 years). In total, 1,375 cervical levels were evaluated in 275 patients. Grade I DD was observed in 16 of the 1375 (1.2%) cervical segment. Grade II DD was observed in 582 of the 1375 (42.3%) cervical segment. Grade III DD was observed in 626 of the 1375 (45.5%) cervical segment. Grade IV DD was observed in 130 of the 1375 (9.5%) cervical segment. Grade V DD was observed in 21 of the 1375 (1.5%) cervical segment (Figure 1).

**Figure 2:**
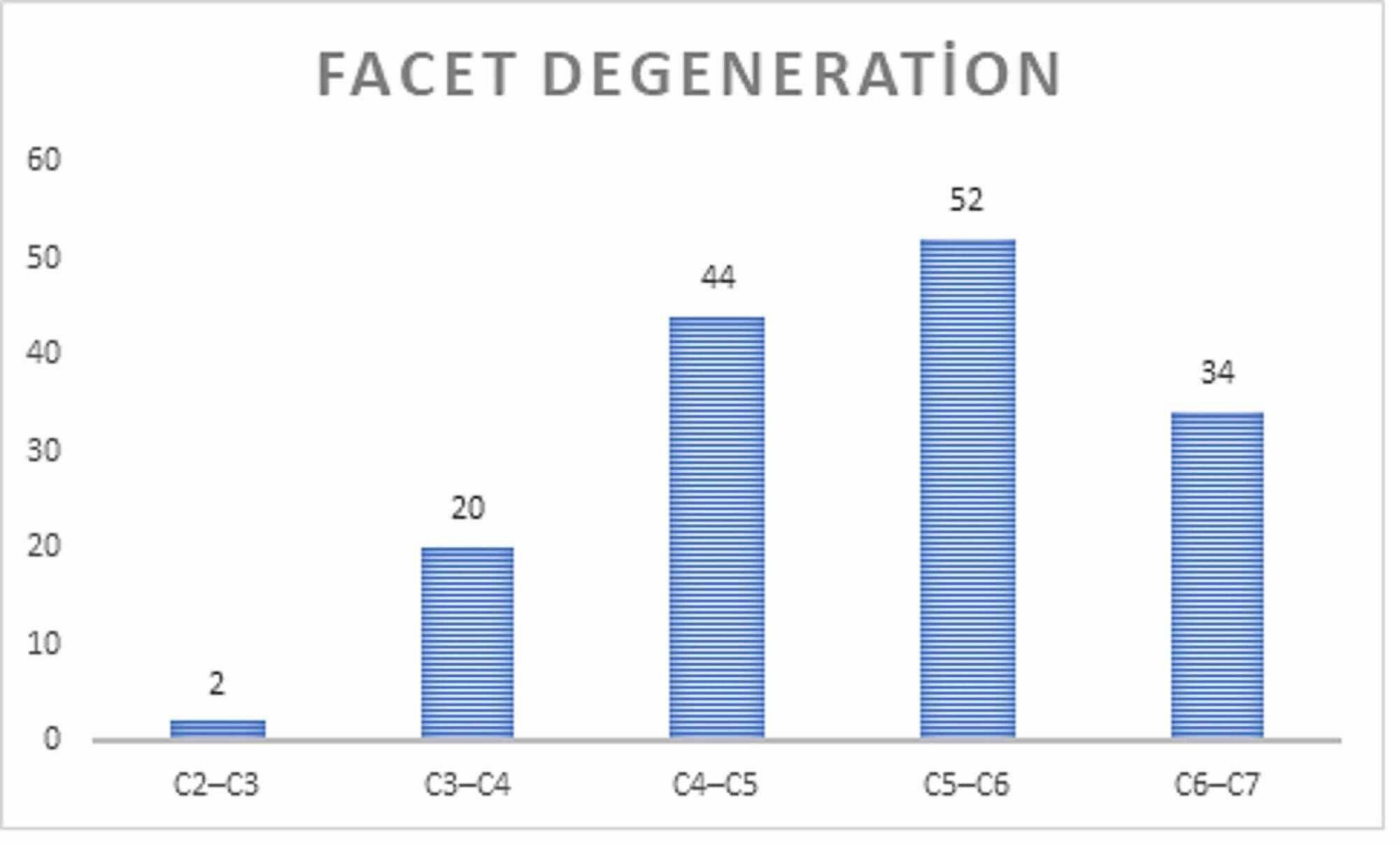
Prevalence of Faset Degeneration at each vertebral level.

**Figure 3:**
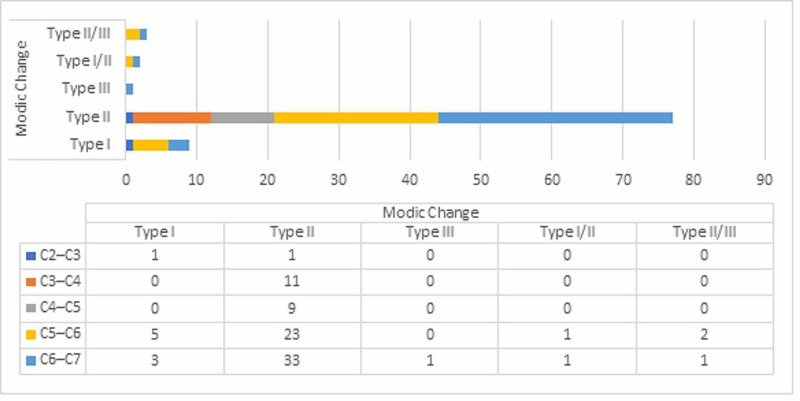
Prevalence of Modic Changes at each vertebral level.

Sixty-six patients had FD (24%) and 75 had MCs (27.2%). FD was observed in 152 of the 1375 (11.1%) cervical segment (Figure 2). FD is most common in the C4-C5 and C5-C6 levels, whereas it is most rarely seen in the C2-C3 level. Among the MC types, MC was observed in 92 of the 1375 (6.7%) cervical segment and type II was the most common; MCs were most commonly seen in the C5-C6 and C6-C7 levels and rarely observed in the C2-C3 level (Figure 3). FD, MCs, and DD of the patients were compared with each other and the results were found to be statistically significant (p < 0.001).

FD, MCs, and DD in each cervical segment of the patients were evaluated separately and compared with each other. The presence of FD in the same segments was associated with any MC at the same level of the cervical spine (p < 0.001). Similarly, when the relationship between the presence of FD and any DD at the same level of the cervical spine was examined, the changes were significant in all segments except for the C2-C3 level (p = 0.410; p < 0.001; p <0.05). When looking at the relationship between the presence of DD and any MC at the same level of the cervical spine, the results were statistically significant at all levels (p < 0.001).

The patients’ mean T1 slope value was 20.85° ± 8.05° (range, 5°-42°), and the mean Cobb angle was 9.61° ± 6.89° (range, 1°-36°). When the relationship between the Cobb angle and T1 slope values was examined, the results were statistically significant (p < 0.001). However, when the angles were compared with the presence or absence of degenerative changes, no statistical relationship was observed.

The following results were obtained when the degenerative changes in each cervical segment of the patients' cervical vertebrae were compared with the Cobb and T1 curvature angular parameters: When compared with the Cobb angle, it was statistically significant with FD at the C4-C5 level (p = 0.04) and with MCs at the C3-C4, C4-C5 and C6-C7 levels (p = 0.013; p = 0.001; p = 0.021). Also, MCs at the C3-C4 level (p = 0.001) and DD at the C2-C3 (p = 0.011) level were statistically significant when compared to T1 slope.

This showed us that the incidence of MCs is higher in increasing angular values at the C3-C4 and C4-C5 levels and decreasing angular values in the C6-C7 level. Likewise, it showed that the incidence of FD is higher in increasing angular values at the C4-C5 level. No statistically significant differences were found in other segments with angular changes.

## Discussion

In this study, we evaluated the relationship between degenerative spinal phenotypes and sagittal balance in patients with neck pain. While the relationship between MCs in the lumbar spine and FD is common in the literature, studies on the relationship between these changes in the cervical spine are scarce [5,12,13].

In the literature, MCs in the cervical vertebrae occur between 5% and 40% and positively correlate with the prevalence of neck pain and DD [14-16]. Hayashi et al. have shown that patients with MCs were more likely to have severe DD at the same segment level (OR, 3.9; 95% confidence interval, 2.42-6.3) compared with those without MC [8]. Additionally, Matsumoto et al. reported that after a 10-year follow-up of 497 asymptomatic patients, the development of MC was positively correlated with various indicators of the progression of DD [7]. There are multiple mechanisms underlying the relationship between DD and MCs [10]. MCs cause degeneration of the endplates, thereby impairing the nutrition of the disc, which is thought to accelerate the degeneration of the disc [8]. In our study, the prevalence of MCs was 27.9%. In addition, when looking at the relationship between the presence of DD and any MC at the same level of the cervical spine, we found that the results were significant at all levels (p < 0.001), consistent with the literature.

Furthermore, the relationship of MCs with FD was examined, and no relationship was observed between FD and MCs at the same level of the cervical spine [7,8,17]. However, Lv et al. showed that the size of MCs was related to cervical FD [5]. With this study, we have seen that MCs, FD, and DD at the same level of the cervical spine are interrelated with each other. This result was similar to the results of a study conducted for the lumbar region [18]. The cervical segment with MCs is prone to DD, and disc herniation leads to a corresponding change in the biomechanical balance of the spine and affects the stability of the cervical spine [8]. This suggests that MCs are risk factors for FD and DD.

Preservation of the integrity of the musculoskeletal system depends on a well-balanced sagittal alignment [19,20]. Sagittal biomechanical instability causes clinical symptoms [21]. As a result of the malalignment of the cervical spine and the decrease in cervical lordosis, the center of gravity of the head shifts. This situation affects the load sharing of the vertebral body and intervertebral discs and increases the axial load on the endplates. This compression force occurring in the endplates extremely sensitive to axial loading may result in MCs of the cervical spine over time [22]. In a recent study in which 100 patients were evaluated, the T1 slope angle was a risk factor for developing MCs due to impaired sagittal balance, especially at the C5-C6 level [23]. In addition, Tong et al. showed that the Cobb angle (cervical lordosis) was smaller in patients with MC than that in those without MC. However, they reported that the presence of MC in all cervical spinal units was severely associated with segmental instability [10]. However, in our study, no significant relationship was found between the Cobb angle or T1 slope values and MC and FD. However, when each cervical segment was examined separately, it showed that the incidence of MC was high in increasing angular values at the C3-C4 and C4-C5 levels and decreasing angular values in the C6-C7 level. It was seen that the MCs at the C3-C4 level were also significant with T1 slope. Our results are consistent with the literature, suggesting that increased axial loading leads to MCs when malalignment of the spine in the sagittal plane occurs [8,10].

## Conclusions

In patients with neck pain, MCs and FD of the cervical endplate mostly occurred at C5/6, then at C6/7 whereas it is most rarely seen in the C2-C3 level. Among the MC types, type II was the most common; MCs were most commonly seen in the C5-C6 and C6-C7 levels. The incidence of MCs is higher in increasing angular values at the C3-C4 and C4-C5 levels and decreasing angular values in the C6-C7 level. Likewise, it showed that the incidence of FD is higher in increasing angular values at the C4-C5 level. MCs increased with decreased cervical curvatuare, increasing disc and facet degeneration, although the causal mechanisms are not clear.
